# Metabolic Responses of Plants to Climate-Induced Stress: A Mass Spectrometry Investigation

**DOI:** 10.1021/acsomega.5c02962

**Published:** 2025-07-09

**Authors:** Matteo Preziati, Enrico Davoli, Antonio Di Guardo, Renzo Bagnati, Elisa Terzaghi, Alice Passoni

**Affiliations:** † Department of Environmental Health Sciences, 9361 Istituto di Ricerche Farmacologiche Mario Negri IRCCS, via Mario Negri 2, 20156 Milano, Italy; ‡ Department of Science and High Technology (DiSAT), 19045 University of Insubria, Via Valleggio 11, 22100 Como, Italy

## Abstract

In the past decade, the increasing global temperature caused by climate change has significantly impacted ecosystems, exposing them to various abiotic stressors, such as drought and salinity, which can alter plant physiology. In response to these abiotic stresses, plants can modify the levels of primary and secondary metabolites and hormones. In this study, we examined the impact of three climate change-related stressors—drought, altered salinity conditions, and acidified watering—on the metabolism of *Lepidium sativum*, using a metabolomic approach based on high-resolution mass spectrometry. MS and MS/MS spectra were analyzed with the Compound Discoverer software for metabolite identification and statistical analysis, while MetaboAnalyst was employed for pathway analysis. Plants exposed to drought stress exhibited the most significant metabolic alterations, with 36 altered metabolites in leaves and 45 in stems. In contrast, plants subjected to salinity stress showed changes in 16 metabolites in leaves and 30 in stems. Finally, plants irrigated with acidified water (pH 3) displayed the fewest altered metabolites, with only 6 in leaves and 2 in stems. The reduced impact of acidified water may be attributed to the soil’s buffering capacity, which could have mitigated the effects of the acidified water. Overall, this study assesses how climate change impacts plant metabolism, paving the way for future research aimed at understanding plant adaptation to climate change, with potential implications for botany, agriculture, and human health.

## Graphical abstract

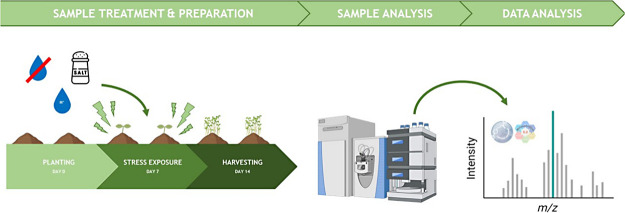

Energy is essential for the economic prosperity of any society. In 2024, about 60% of global energy consumption came from coal, oil, and natural gas,^1^ as fossil fuels still provide the majority of the world’s energy needs. The combustion of these fossil fuels releases greenhouse gases (GHGs), such as carbon dioxide (CO_2_), methane (CH_4_), and nitrous oxide (N_2_O),^2^ which contribute significantly to climate change. According to the AR6 report by the Intergovernmental Panel on Climate Change, atmospheric concentrations of CO_2_, CH_4_, and N_2_O in the atmosphere increased by 47%, 156%, and 23%, respectively, starting from 1750 to 2019.^3^ In 2024, energy combustion contributed 34684 million tonnes of carbon dioxide equivalent (MtCO_2_eq).^1^ The increasing emission of GHGs contributed to the average global temperature increase of +1.1 °C in the decade 2011–2020 as compared to 1850–1900.^2^

These temperature changes had a major impact on both ecosystems and human health; for instance, a temperature increase of +1 °C above the monthly average in the United States and Mexico has been associated with an increase in mental illness^4^ and exposure to infectious diseases.^5^ The global economy is also affected:^6^ countries such as the Democratic Republic of the Congo, Ethiopia, Nigeria, Burkina Faso, Uganda, and China suffered from severe droughts.^7^ As a consequence of these rapid changes related to climate change, ecosystems are increasingly exposed to abiotic stressors, such as altered salinity, drought, and soil acidification. Climate change drives these stressors through mechanisms such as increasing global temperatures, shifts in precipitation patterns, increased frequency and intensity of extreme weather events, and elevated atmospheric CO_2_ levels. Particularly, drought and salinity have been demonstrated to have a substantial impact on plant physiology;^8^ they frequently happen concurrently and, depending on the severity and duration of the stress, as well as the stage of the plant life cycle, cause intricate metabolic and transcriptional reactions.^9,10^

Plants possess adaptive mechanisms to cope with environmental stressors, such as drought, often through alterations in metabolic pathways. These changes lead to significant variations in the levels of primary and secondary metabolites, as well as plant hormones. Primary metabolites, such as sugars, lipids, and amino acids, are critical for growth and development, and they are highly conserved across species.^11,12^ Secondary metabolites, on the other hand, play diverse roles such as interacting with the environment and enhancing resistance to stress. Examples include phenols, flavonoids, nitrogen-containing compounds, and terpenes. Hormones like jasmonic acid (JA) and abscisic acid (ABA) regulate many of these metabolic processes and play key roles in the synthesis of primary and secondary metabolites.^11^

Drought, one of the most impactful abiotic stressors, is exacerbated by extreme temperature fluctuations linked to climate change.^8,13^ While the average global temperature rise is projected to be around 1.5 °C, localized extreme events could lead to significant fluctuations. Despite various physiological adaptations, such as stomatal closure, plants may still struggle to cope with the increasing frequency and severity of these events. Under drought conditions, plants reduce transpiration by accumulating abscisic acid in guard cells, which triggers stomatal closure to conserve water.^14^ They also adjust their metabolism by accumulating osmoprotectants like mannitol, reducing reactive oxygen species (ROS) production, and stabilizing enzyme structures to prevent protein damage.^15,16^ Similarly, excessive soil salinity limits water and nutrient availability, causing hypertonic stress, which can lead to plant death. Plants subjected to high salinity conditions respond by producing metabolites such as amino acids, polyphenols, and jasmonic acid to mitigate stress.^8,17^

Another major abiotic stressor affecting plant health is acid rain, caused by air pollution, which affects soil properties, such as nutrient availability, decrease in microbial biomass, diversity, pH, bioavailability of toxic metals, such as aluminum, and, consequently, plant growth. In particular, the aboveground parts of the plant could show a reduction in fresh weight, photosynthetic activity, and structural and cellular damage.^18^ Additionally, acid rain can have either positive or negative effects on belowground biomass, depending on its frequency and pH. However, the soil buffering capacity can mitigate the effects of infrequent acid rainfall. In addition to that, acid rain can have a fertilizer effect by supplying nitrogen and sulfur, as long as these are infrequent and not continuous.^19,20^

Despite existing research, much of what we know about metabolic alterations caused by abiotic stressors is fragmented and often limited to a few metabolic pathways. A key example is the role of the tricarboxylic acid (TCA) cycle in plant responses to stress, as many metabolic intermediates of this cycle, such as citric acid, serve as precursors for secondary metabolite synthesis.^21^ However, numerous crosstalks between signaling and metabolic pathways in plants affected by abiotic stressors still remain unclear and unsolved. At the same time, increasing temperatures and adverse weather are pressing our agricultural system to develop new strategies to address problems such as crop reduction and soil infertility. Given the demanding challenges posed by rising temperatures and extreme weather events, the agricultural sector must develop new strategies to address crop reduction and soil infertility. In this context, the present study aims to evaluate the metabolic response of *Lepidium sativum* to abiotic stressors related to climate change, specifically focusing on drought, salinity, and acidified water. We used *Lepidium sativum* due to its well-established use as a model organism in the ecotoxicological assay. Moreover, it is an edible plant for human use.^22,23^ By analyzing stems and leaves separately, we aim to identify metabolic signals associated with plant resilience under these conditions. This work represents the first study to examine the metabolic responses of *L. sativum* to climate-change-related stressors using high-resolution mass spectrometry and the Compound Discoverer software for detailed MS data analysis. The insights gained here have the potential to inform future research in botany, agriculture, and human health.^24^

## Results and Discussion

### Definition of the Watering Conditions

Optimal watering conditions for experiments were determined by comparing irrigation with Milli-Q and tap water on plant metabolites. As shown in Figure 1, our metabolic analysis revealed that no metabolites were significantly altered in either tissue type. This was also supported by the results of shoot biomass and height, which did not show statistically significant differences (*p* > 0.05) (Figure 2). Based on these findings, we decided to use Milli-Q water for all subsequent analyses.

**Figure 1:**
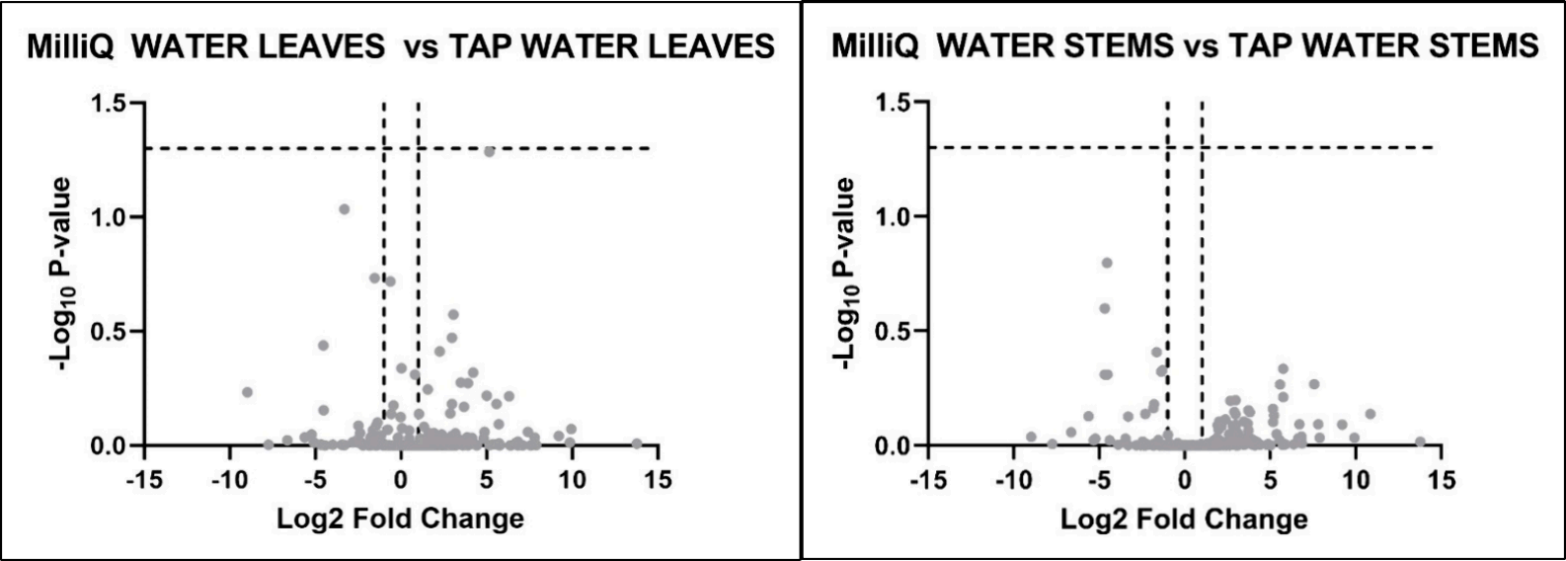
Volcano plot of identified metabolites in the leaves (left) and stems (right) of *Lepidium sativum* irrigated with Milli-Q water compared to tap water. The *x*-axis represents the log2 fold change (FC) of metabolites between the two watering conditions, while the *y*-axis indicates the −log10 of the *p*-values from statistical analyses. The dotted horizontal and vertical lines indicate the threshold for statistical significance (*p* < 0.05; FC > 1).

**Figure 2:**
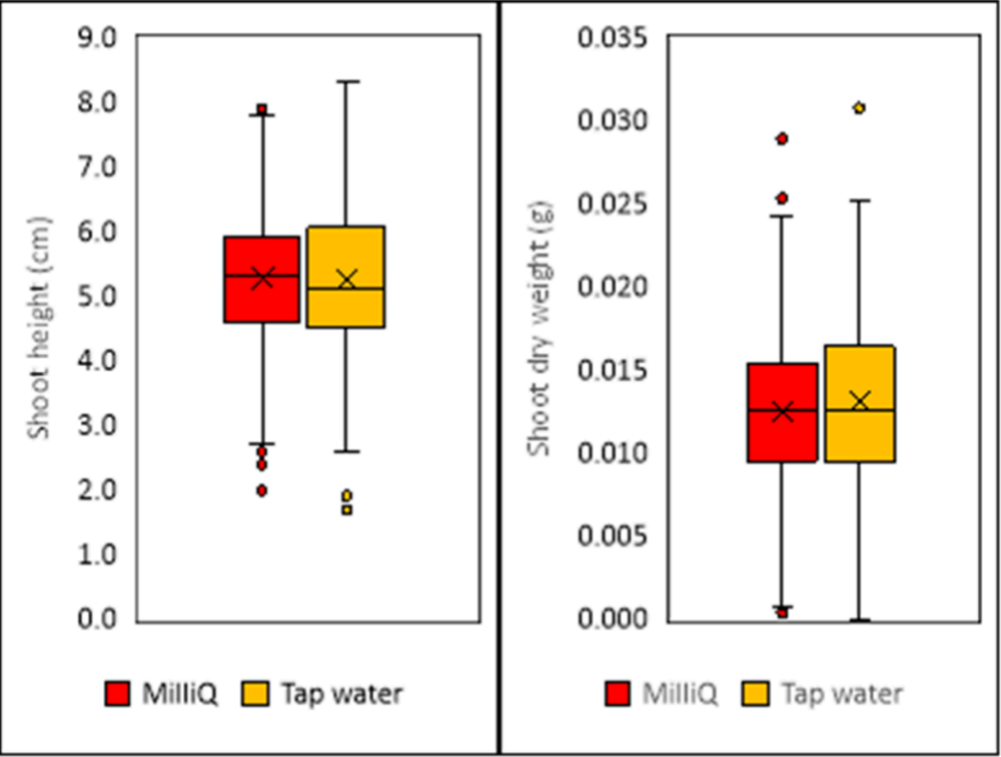
Shoot height and dry weight of controls irrigated with Milli-Q (red) and tap water (orange).

### Selection of the Abiotic Stressors Related to Climate Change

Abiotic stressors affecting plants include temperature fluctuations, soil salinity and pH variations, water scarcity, radiation exposure, chemical pollutants, and nutrient depletion.^8^ Drought, salinity, and low pH, correlated with climate change, were studied due to their simultaneous occurrence and impact on plants.^9,10^

### Stressor Effect on Plant Growth

The ecological traits of *L. sativum* exposed to different treatments are shown in Figure 3 and Table 1. While shoot height did not show a statistically significant difference between the control and the treatments, the selected stressors significantly affected shoot dry biomass and leaf number with respect to the control. More specifically, drought, high salinity, and acidity negatively influenced shoot dry biomass, while leaf number seemed to be influenced just by drought and high salinity.

**Figure 3:**
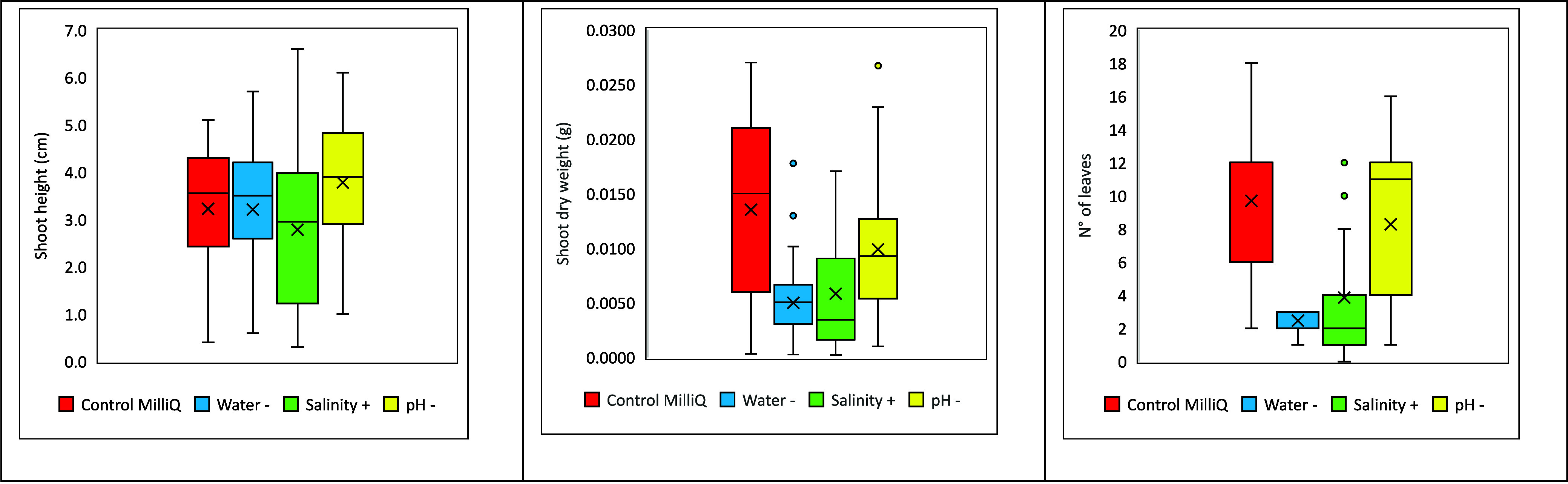
Shoot height, dry weight, and number of leaves of control and treatments.

**Table 1:** ANOVA and Tukey’s Test Result Summary

traits	*F*-ratio	*p*-value	pairwise comparisons
height	7.15	0.000121	control = water–; control = salinity+; control = pH 3; water– = salinity+; water– ≠ pH 3; salinity+ ≠ pH 3
dry weight	34.72	<0.00001	control ≠ water–; control ≠ salinity+; control ≠ pH 3; water– = salinity+; water– = pH 3; salinity+ = pH 3
no. of leaves	62.25	<0.00001	control ≠ water–; control ≠ salinity+; control = pH 3; water– = salinity+; water– ≠ pH 3; salinity+ ≠ pH 3

### Stressor Effect on Plant Metabolism

The selected stressors differently affected plant metabolism. Table 2 summarizes the number of significantly altered plant metabolites in the three treatments with respect to the controls and the altered metabolic pathways. The complete list of all identified metabolites in leaf and stem samples is provided in Tables S1 and S2, respectively. Briefly, the metabolomic results are in good agreement with the trend in plant ecological traits. Drought seemed to be the most important treatment in influencing plant traits and metabolism (in terms of the number of significantly altered plant metabolites and metabolic pathways) (Table 3).

**Table 2:** Summary of Altered Metabolites and Metabolic Pathways

	no. of metabolites			
	leaves	stem	no. of pathways	type of pathway
water–	36	45	1	lysine degradation
salinity+	16	30	2	lysine degradation, glycine, serine and threonine metabolism
pH 3	4	2	0	

**Table 3:** Summary of Altered Plant Traits, Metabolites, and Metabolic Pathways

	metabolomic			plant traits		
	no. of metabolites					
	leaves	stem	no. of pathways	shoot height	shoot dry biomass	no. of leaves
water–	36	45	1	no	yes	yes
salinity+	16	30	2	no	yes	yes
pH 3	4	2	0	no	yes	no

### Drought Effect on Plant Metabolism

#### Leaves

Seven days of drought conditions resulted in the identification of 36 significantly altered metabolites in *Lepidium sativum* leaves (Figure 4). Metabolic pathway analysis highlighted the lysine degradation pathway as the only altered pathway in plants grown in drought conditions (−log­(*p*) = 3.0366; FDR = 0.044121) (Table 4). A complete list of all identified metabolites in drought-treated leaf samples is provided in Table S3.

**Figure 4:**
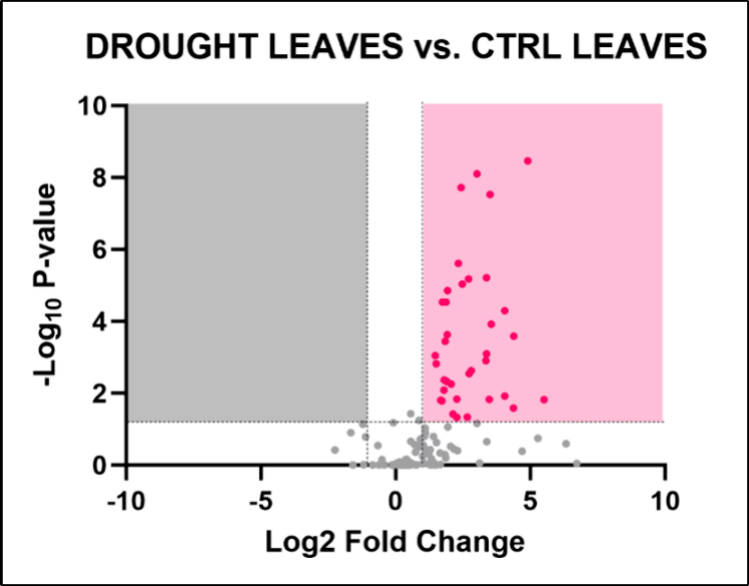
Volcano plot illustrating the identified metabolites in *Lepidium sativum* leaves grown under drought conditions. The plot displays the statistical significance (−log­(*p*)) versus the magnitude of change (fold change) of each metabolite, highlighting the metabolites that are significantly altered in response to drought stress. Metabolites above the threshold line indicate significant differences compared to control conditions, with 36 metabolites identified as significantly altered. The dotted horizontal and vertical lines indicate the threshold for statistical significance (*p* < 0.05; FC > 1).

**Table 4:** Metabolic Pathway Analysis Results Obtained from MetaboAnalyst, Including the Metabolic Pathway, −Log­(*p*), FDR, and Coverage (Defined as the Number of Metabolites Identified within the Metabolic Pathway under Examination)

altered metabolic pathways			
metabolic pathway	–log(*p*)	**FDR**	**coverage**
*lysine degradation*	3.0366	0.044121	3/18

Khan et al. examined the growth of *Cicer arietinum* plant leaves under drought stress using high-resolution mass spectrometry. Their findings revealed that drought conditions led to an accumulation of specific metabolites, including allantoin and various amino acids, such as arginine, proline, tryptophan, tyrosine, histidine, and isoleucine. Except for tyrosine and isoleucine, in our study, we observed an increasing level in the total content of amino acids and amino-acid-related metabolites.

In contrast, the concentrations of purine components, like guanine and adenosine, along with amino acids, such as alanine and phenylalanine, as well as metabolites, including gamma-aminobutyric acid, choline, glucosamine, and aspartic acid, were found to be lower in the control group under nonstress conditions.^25^

However, in our study, the levels of guanine and phenylalanine were detected at higher levels compared to the control group. In contrast, the other metabolites resulted in not being dysregulated or were not detected.

In leaves, we observed higher levels of valine but not leucine and isoleucine. Together, these amino acids are known as branched-chain amino acids (BCAAs). Shim et al. studied the effects of water deficit conditions on rice (*Oryza sativa*) and found that the levels of BCAAs significantly increased due to the enhanced expression of the OsDIAT gene in both leaves and roots. This increase in BCAAs was indicative of the plant’s response to drought stress, suggesting that OsDIAT plays a crucial role in amino acid metabolism during such conditions. Furthermore, similar changes in BCAA levels were also observed in rice leaves subjected to high salinity stress and low temperatures, indicating a broader role for OsDIAT in abiotic stress responses.^26^

#### Stems

In the stems of plants grown under water deficiency conditions, a significant alteration of 45 different metabolites was observed (Figure 5). This metabolic shift mirrors the changes noted in leaves, where drought conditions also influenced the lysine degradation pathway (−log­(*p*) = 2.7585; FDR = 0.083701) (Table 5).

**Table 5:** Metabolic Pathway Analysis Results Obtained from MetaboAnalyst, Including the Metabolic Pathway, −Log­(*p*), FDR, and Coverage (Defined as the Number of Metabolites Identified within the Metabolic Pathway under Examination)

altered metabolic pathways			
metabolic pathway	–log(*p*)	**FDR**	**coverage**
*lysine degradation*	2.7585	0.083701	3/18

**Figure 5:**
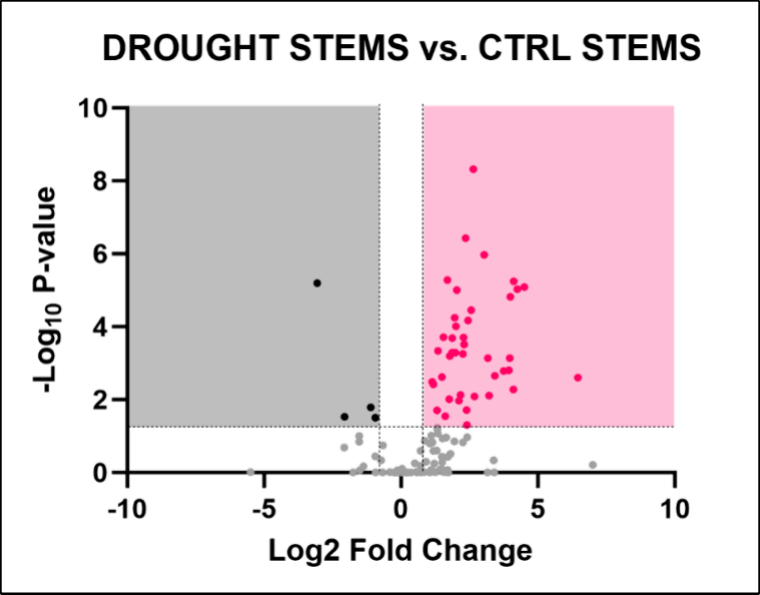
Volcano plot illustrating the identified metabolites in *Lepidium sativum* stems grown under drought conditions. The plot displays the statistical significance (−log­(*p*)) versus the magnitude of change (fold change) of each metabolite, highlighting the metabolites that are significantly altered in response to drought stress. Metabolites above the threshold line indicate significant differences compared to control conditions, with 45 metabolites identified as significantly altered. The dotted horizontal and vertical lines indicate the threshold for statistical significance (*p* < 0.05; FC > 1).

A complete list of all identified metabolites in drought-treated stem samples is provided in Table S4.

In the drought-treated group, levels of l-lysine, saccharopine, and aminoadipic acid were significantly higher in both stems and leaves compared to the control group (Figure 6). It is well reported in the literature that plants grown in drought conditions exhibit an increased activity of the LKR/SDH enzyme, which is crucial for lysine metabolism and related pathways.^27^ The elevated levels of abscisic acid (ABA) and jasmonic acid (JA), hormones released in response to stress, are responsible for the transcriptional activation of the genes encoding the LKR/SDH and AASADH enzymes. This transcriptional process appears to be further enhanced by the high levels of ABA and JA, which can activate these enzyme genes. Notably, plant species resistant to drought tend to have higher concentrations of aminoadipic acid and saccharopine, two metabolites produced by LKR/SDH, serving as osmoprotectants.^28,29^

**Figure 6:**
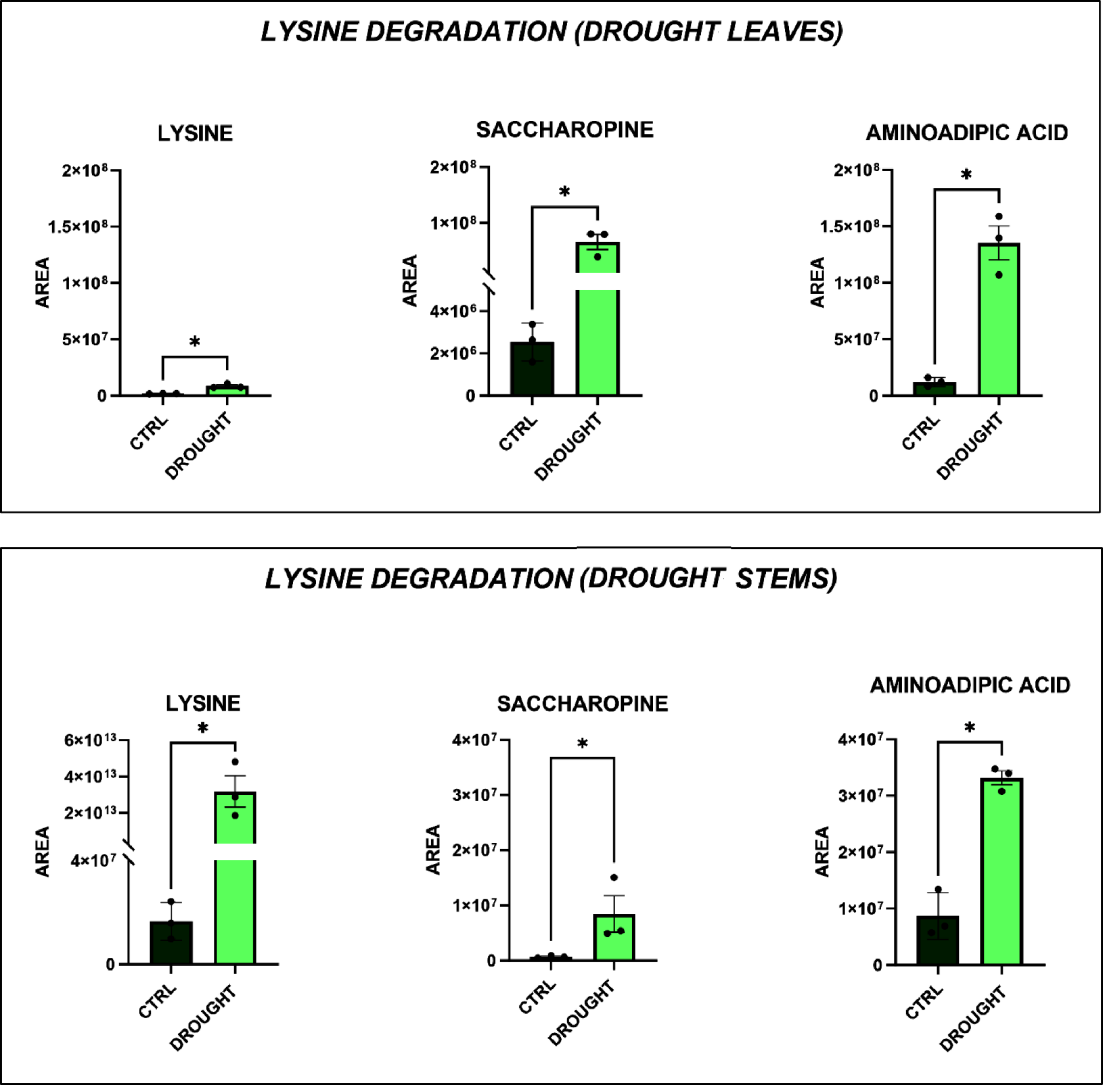
Significantly altered metabolic pathways in *Lepidium sativum* leaves (top) and stems (bottom) under drought conditions. Each bar represents the average peak area for each metabolite, with error bars indicating the standard deviation (*p-value <0.05; one-way ANOVA, followed by Tukey’s post hoc test).

These findings underscore the complex interplay between hormonal signaling and metabolic pathways in plants grown under drought stress. Our data are consistent with the literature; as a matter of fact, the level of JA was found to be upregulated in both leaves and stems, suggesting that JA may play a significant role in the plant’s systemic response.

You et al. demonstrated that drought-tolerant wheat genotypes exhibit significantly higher levels of nonenzymatic antioxidants in their roots compared to drought-sensitive species. This finding highlights the enhanced capacity of drought-tolerant varieties to mitigate oxidative stress under water-limited conditions. In particular, the study identified three anthocyanidin monomers—malvidin 3-*O*-glucoside, cyanidin 3,5-*O*-diglucoside, and delphinidin 3-*O*-glucoside—along with four flavonoids: kaempferol 3-*O*-galactoside, quercetin, homoeriodictyol, and hesperetin. These compounds are known for their antioxidant properties, which help protect plant tissues from oxidative damage caused by reactive oxygen species (ROS) that accumulate during drought stress.^29^ In addition, the level of ABA was upregulated in both genotypes; however, it was significantly more elevated in drought-tolerant species. Furthermore, several metabolites, such as sinapyl alcohol, *p*-coumaryl alcohol, sinapic acid, caffeic acid *O*-glucoside, and *p*-coumaraldehyde, were found to accumulate in drought-tolerant species while being downregulated in drought-sensitive varieties.

Al-Huqail et al. observed that *Ocimum basilicum* plants exposed to water stress exhibited an accumulation of several key metabolites, including glycine-betaine, proline, malondialdehyde, and total phenolic compounds. This accumulation suggests that these metabolites play a significant role in the plant’s response to drought conditions, helping to mitigate oxidative stress and maintain cellular integrity.^30^

### Salinity Effect on Plant Metabolism

#### Leaves

Altered salinity conditions caused a significant alteration of 16 metabolites in leaves (Figure 7), mainly amino acids and their derivatives and 2 phenolic compounds. Nevertheless, the metabolic pathway analysis using MetaboAnalyst did not reveal any significantly altered pathways. A complete list of metabolites identified in leaf samples treated with high-salinity water is provided in Table S5. A previous study on leaves of *Vitis vinifera* demonstrates that salt stress induces the expression of key genes involved in the ABA signaling and MAPK signaling pathways. Additionally, the abundance of various organic acids rose, along with the upregulation of genes encoding ion transporters. On the other hand, the content of the majority of the sugar metabolites decreased.^31^

**Figure 7:**
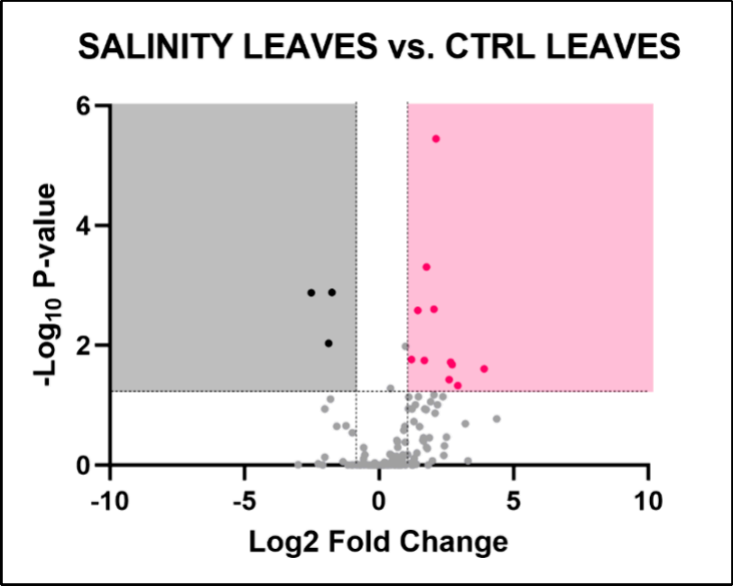
Volcano plot illustrating the identified metabolites in *Lepidium sativum* leaves grown under altered salinity conditions. The plot displays the statistical significance (−log­(*p*)) versus the magnitude of change (fold change) of each metabolite, highlighting the metabolites that are significantly altered in response to drought stress. Metabolites above the threshold line indicate significant differences compared to control conditions, with 16 metabolites identified as significantly altered. The dotted horizontal and vertical lines indicate the threshold for statistical significance (*p* < 0.05; FC > 1).

#### Stems

Thirty metabolites were significantly dysregulated in stems from plants grown in altered salinity conditions (Figure 8), resulting in the alteration of two metabolic pathways: Lysine degradation (−log­(*p*) = 3.5101; FDR = 0.01483) and glycine, serine, and threonine metabolism (−log­(*p*) = 2.7149; FDR = 0.061702) (Table 6).

**Figure 8:**
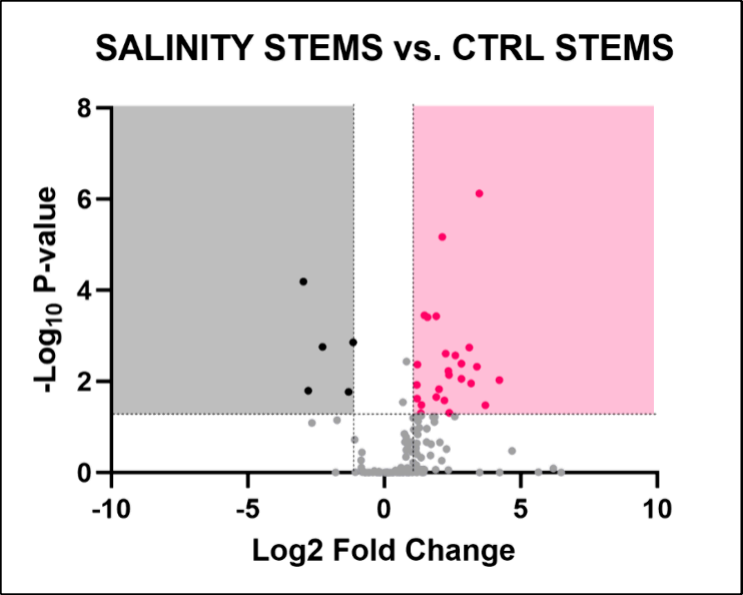
Volcano plot illustrating the identified metabolites in *Lepidium sativum* stems grown under altered salinity conditions. The plot displays the statistical significance (−log­(*p*)) versus the magnitude of change (fold change) of each metabolite, highlighting the metabolites that are significantly altered in response to drought stress. Metabolites above the threshold line indicate significant differences compared to control conditions, with 30 metabolites identified as significantly altered. The dotted horizontal and vertical lines indicate the threshold for statistical significance (*p* < 0.05; FC > 1).

**Table 6:** Metabolic Pathway Analysis Results Obtained from the Analysis Using MetaboAnalyst (Metabolic Pathway, −Log­(*p*), FDR, Coverage Understood as Metabolites Identified within the Metabolic Pathway under Examination)

altered metabolic pathways			
metabolic pathway	–log(*p*)	**FDR**	**coverage**
*lysine degradation*	3.5101	0.01483	3/18
*glycine, serine, and threonine metabolism*	2.7149	0.061702	3/33

A complete list of metabolites identified in stem samples treated with high-salinity water is provided in Table S6.

l-Lysine, aminoadipic acid, and saccharopine were significantly altered in stems from the salinity-treated group in comparison to the control group, as previously seen in the case of drought conditions (Figure 10). Similarly, aminoadipic acid and saccharopine serve as osmoprotectants through the same mechanism, helping to stabilize cellular functions under stress conditions. Additionally, plants can enhance their response to salt stress by using lysine to build up carbohydrates, which is crucial for maintaining metabolic balance under saline conditions.^32^ One important pathway involves the conversion of lysine to cadaverine through a decarboxylation reaction, known as the cadaverine pathway. This process yields cadaverine, a molecule classified as a polyamine. Cadaverine possesses a positive charge, which contributes to its role as an osmoprotectant. Its positive charge helps stabilize cellular structures and mitigate the effects of osmotic stress, thereby supporting plant resilience in high-salinity environments.^33^

The glycine, serine, and threonine metabolic pathway counted 33 metabolites, with 3 of them dysregulated, in our results. Specifically, l-serine and l-threonine were significantly downregulated, while l-tryptophan was significantly upregulated (Figure 9). These findings contradict observations in the literature, which typically indicate that plants exposed to osmotic stress exhibit higher levels of both tryptophan and serine. l-serine is also implicated in the synthesis of the osmoprotectant glycine-betaine, although we cannot provide sufficient evidence for this relationship due to the lack of data regarding glycine-betaine levels in our analysis (Figure 10).^34^

**Figure 9:**
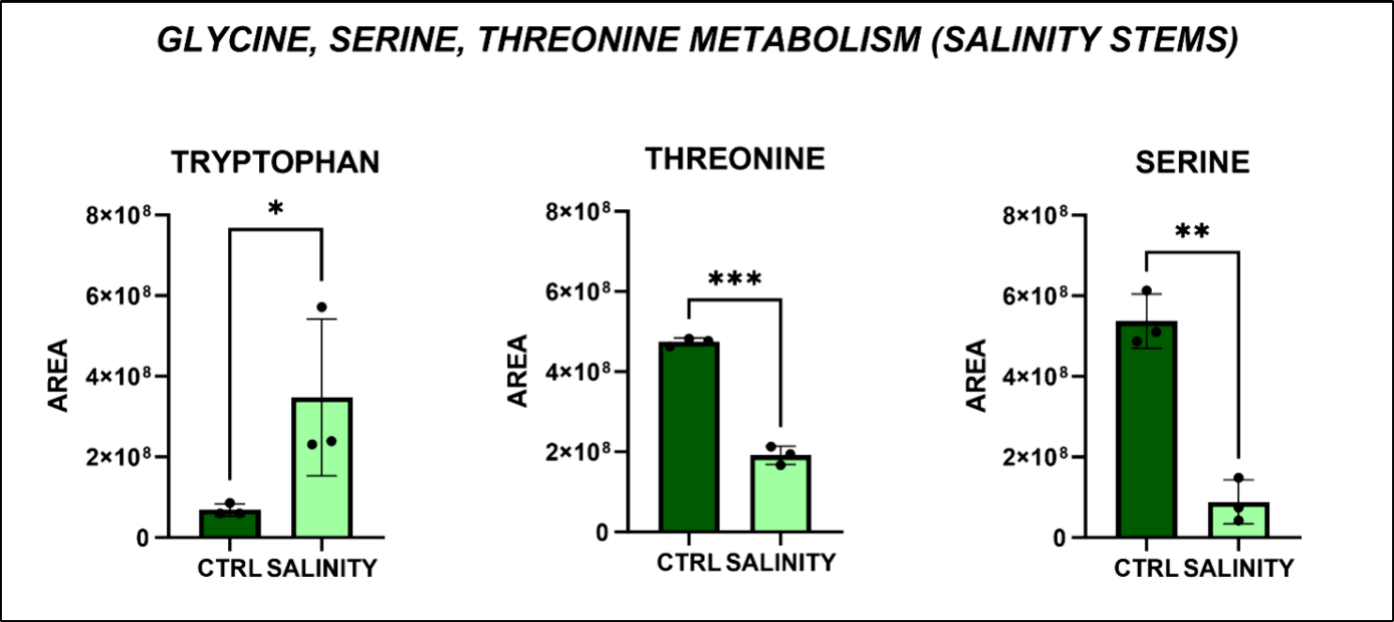
Significantly altered metabolic pathways in *Lepidium sativum* and stems under altered salinity conditions. Each bar represents the average peak area for each metabolite, with error bars indicating the standard deviation (*p*-value: * < 0.05; **<0.01; ***<0.005; one-way ANOVA, followed by Tukey’s post hoc test).

**Figure 10:**
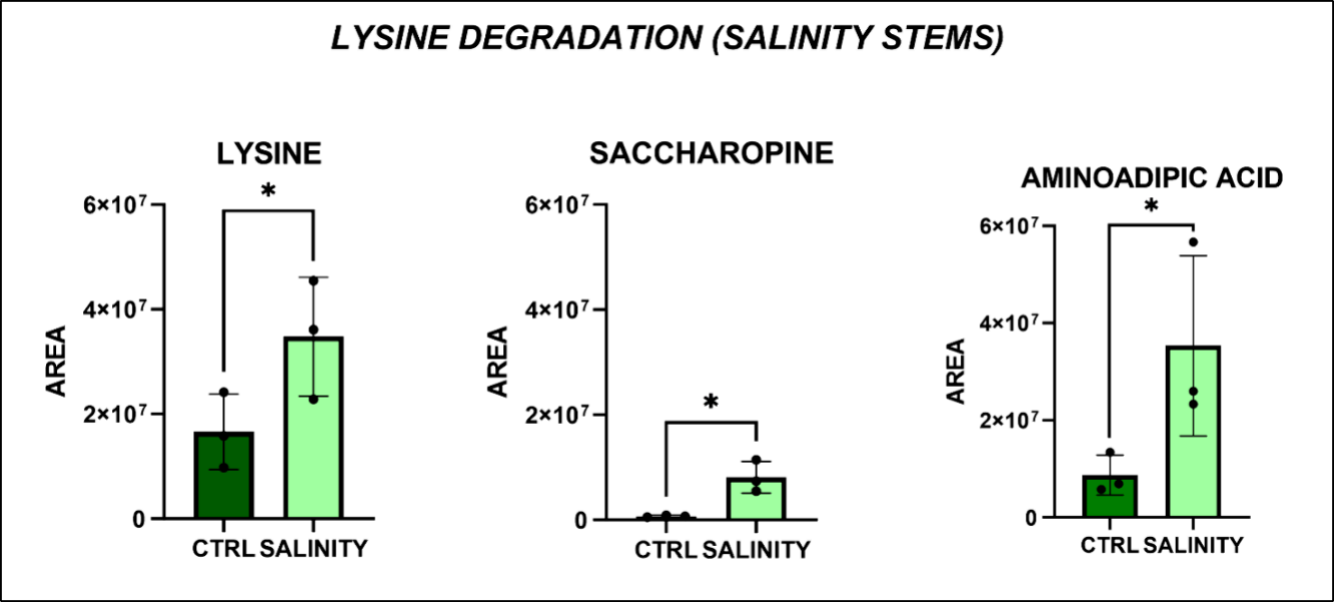
Significantly altered metabolic pathways in *Lepidium sativum* and stems under salinity conditions. Each bar represents the average peak area for each metabolite, with error bars indicating the standard deviation (*p*-value: * < 0.05; one-way ANOVA, followed by Tukey’s post hoc test).

On the other hand, threonine serves as a precursor of BCAAs, which can help plants cope with osmotic stress and provide an alternative source of energy. It is plausible to hypothesize that the decrease in threonine levels may facilitate the synthesis of other metabolites that assist the plant in managing stress. However, the lack of data on threonine-derived metabolites limits the confirmation of our hypothesis.

The analysis carried out on both leaves and stems highlighted an increased level in phenolic compounds, such as syringic acid, ferulic acid, 4-coumaric acid, and gingerol. In a previous study, performed on *Lepidium sativum* exposed to drought and salt stress, the total content of phenolic compounds showed an increment, while the total content of flavonols decreased.^35^

Notably, it is well documented that JA levels increase in plants exposed to salinity conditions,^8^ indicating that this hormone is part of a broader adaptive strategy employed by plants to cope with multiple abiotic stressors. In this case, JA was detected in both tissues of plants treated with high-salinity watering; however, it was not found to be dysregulated. This may be due to the fact that the duration or the intensity of the applied stress is not stressful enough.

In stems of *Hibiscus cannabinus* grown under salinity stress, it was observed an increased level of maltose. This accumulation of maltose is likely related to the plant’s ability to manage osmotic responses, helping to maintain cellular turgor and stability in the face of elevated salt concentrations. Conversely, there was a reduction in the levels of indole acetic acid (IAA), a key plant hormone involved in growth and development. Additionally, abscisic acid and gibberellin A4 were detected in greater amounts with respect to the normal condition group. In contrast, jasmonic acid and salicylic acid were detected in lower quantities compared to the normal group.^36^

### Effect of Acidified Water on Plant Metabolism

#### Leaves

This stress condition caused the significant dysregulation of 7 metabolites: l-histidine, saccharopine, leucylproline, syringic acid, gingerol, l-phenylalanine, and L-tryptophan (Figure 11). Metabolic pathway analysis did not highlight any altered pathway. It is possible that the buffering capacity of the soil may have reduced the stress.

**Figure 11:**
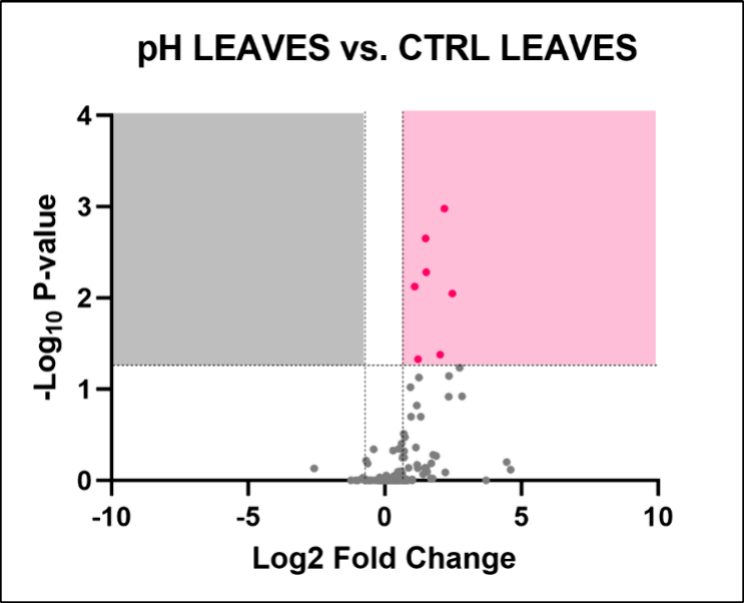
Volcano plot illustrating the identified metabolites in leaves of *Lepidium sativum* watered with acidified water. The plot displays the statistical significance (−log­(*p*)) versus the magnitude of change (fold change) of each metabolite, highlighting the metabolites that are significantly altered in response to drought stress. Metabolites above the threshold line indicate significant differences compared to control conditions, with 30 metabolites identified as significantly altered. The dotted horizontal and vertical lines indicate the threshold for statistical significance (*p* < 0.05; FC > 1).

A complete list of metabolites identified in leaf samples treated with acidified water is provided in Table S7.

#### Stem

Similarly, no altered metabolic pathways were identified in the stems. The analysis reported only two significantly altered metabolites: (*S*)-2-propyl piperidine and syringic acid (Figure 12). A complete list of metabolites identified in stem samples treated with acidified water is provided in Table S8.

**Figure 12:**
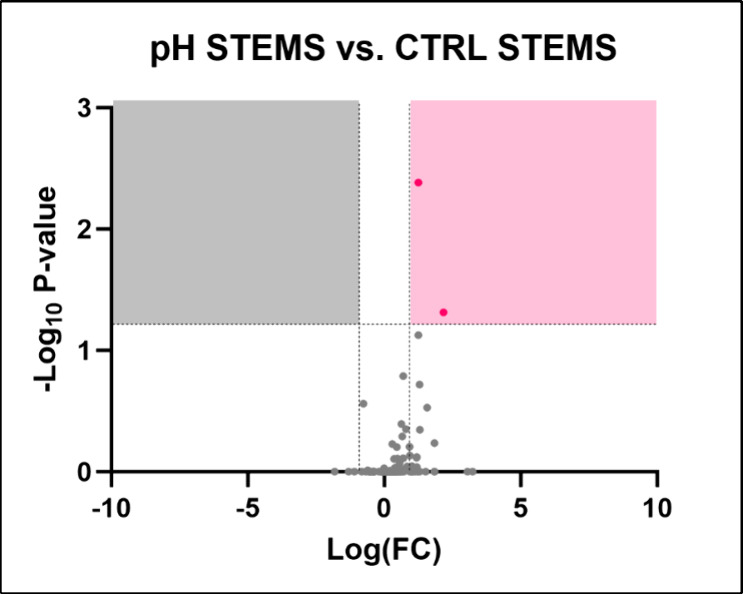
Volcano plot illustrating the identified metabolites in stems of *Lepidium sativum* watered with acidified water. The plot displays the statistical significance (−log­(*p*)) versus the magnitude of change (fold change) of each metabolite, highlighting the metabolites that are significantly altered in response to drought stress. Metabolites above the threshold line indicate significant differences compared to control conditions, with 30 metabolites identified as significantly altered. The dotted horizontal and vertical lines indicate the threshold for statistical significance (*p* < 0.05; FC > 1).

In recent years, there has been growing interest in dipeptides and tripeptides due to their potential roles in cell metabolism and signaling. Dipeptides are primarily formed as a result of protein degradation, which can occur from proteins that are no longer needed by the plant or from those with incorrect structures. Stress conditions may induce an increased production of di- and tripeptides, as plants degrade proteins to obtain energy and catabolites to cope with stress.^37^ The elevated levels of lysine observed under drought and altered salinity conditions may stem from protein degradation, which can subsequently be utilized to produce compounds such as saccharopine, aiding in the management of osmotic stress.^38^ Additionally, certain stress conditions might negatively impact specific proteins, leading to a decrease in enzymatic activity and alterations in the protein structure. Consequently, elevated levels of peptide fragments could indicate the heightened catabolic activity associated with misfolded or inactive proteins.

Overall, our study aimed to evaluate the effects of specific abiotic stress conditions on plant metabolism by using a high-resolution mass spectrometry approach. The selected stress conditions—drought, salinity, and acidified soil—were designed to mimic climate change effects. Our study demonstrated how a single abiotic stressor can induce an alteration in a plant’s metabolism. Among the selected stress conditions, drought was the most impactful one on plant metabolism, affecting both stem and leaves in a similar manner. Specifically, drought stress significantly altered 36 metabolites in leaves and 45 metabolites in stems. These altered metabolites belonged to various compound classes, including flavonoids, fatty acids, phytohormones, and nucleotides. However, the total number of altered metabolites was lower under salinity stress, with 16 in leaves and 30 in stems. The buffering action of salt in the soil mitigated the impact of acidified watering, which did not significantly affect the plant metabolism. Pathway analysis highlighted that the lysine degradation pathway was dysregulated in plants exposed to both drought and salinity stress. As a matter of fact, the lysine degradation pathway, as previously discussed, is involved in the plant response to osmotic and salt stress. Knowing the metabolites that are altered by the single stress could help for further studies that aim to combine both stress for a more complete holistic understanding of plant response to stress. While this observation aligns with previous studies implicating this pathway in responses to osmotic and salt stress, it is important to note that our study did not directly measure pathway activity or gene expression. Thus, any mechanistic interpretation should be considered speculative and warrants further validation.

In conclusion, our study highlighted the potential of a metabolomic approach to assess the effects of abiotic stressors related to climate change on plant metabolism. In this study, we evaluated the impact of a single abiotic stress to understand and define the metabolic responses of the plants to a single metabolic stressor. This laid the groundwork for further research, including the combination of numerous stressors. This study presents a novel application of high-resolution mass spectrometry to comprehensively assess the metabolic responses of *Lepidium sativum* to various abiotic stressors, specifically drought, salinity, and acidified soil. High-resolution mass spectrometry provides a wealth of data that offer valuable insights into plant responses to abiotic stressors. Our findings pave the way for further analyses that integrate information about plant responses to climate change, including proteomics and genomics. These combined approaches can more effectively elucidate the functioning of individual metabolic pathways and various mechanisms that plants activate in response to environmental stress.

## Materials and Methods

### Chemicals and Reagents

D5-glutamic acid, D8-phenylalanine, and D7-propranolol were obtained from Merck Life Science S.r.l. and used as internal standards (IS). All solvents were of LC-MS grade: acetonitrile, methanol, formic acid, ammonium formate (Carlo Erba Reagents), and water (in-house Milli-Q apparatus). D7-propranolol was dissolved in methanol to a 100 mg/mL concentration. Standard solutions of D5-glutamic acid and D8-phenylalanine were prepared in Milli-Q water at a 1 mg/mL concentration. Each standard solution was then diluted 1:10 with a mixture of water:methanol (50:50%, v/v). Each pot for growing plants was composed of 35 g of sand and 35 g of compost; seeds were bought from Germisem.

### Sample Treatment

Twenty *Lepidium sativum* seeds were sowed in each pot, grown under various stress conditions, and treated as described in Figure 1. The conditions in the growth chamber were the following: 21 °C; light conditions: 2000 lx; 14 h light/10 h dark; soil water condition: 40%. Each condition had six replicates, which were subsequently combined into triplicate. Seven days after sowing, the plants were divided into five groups. One group served as a control (watered with Milli-Q water), while another group was watered with tap water to investigate potential differences compared to Milli-Q water. The remaining three groups were subjected to different abiotic stressors related to climate change for 7 days: high-salinity watering (NaCl at 20 g/L), acidified watering (pH 3), and no watering (drought conditions). Fourteen days after sowing, plants were harvested, measured for morphofunctional trait determination (see Figure 13), stored at −20 °C, and prepared for metabolomic analysis.

**Figure 13:**
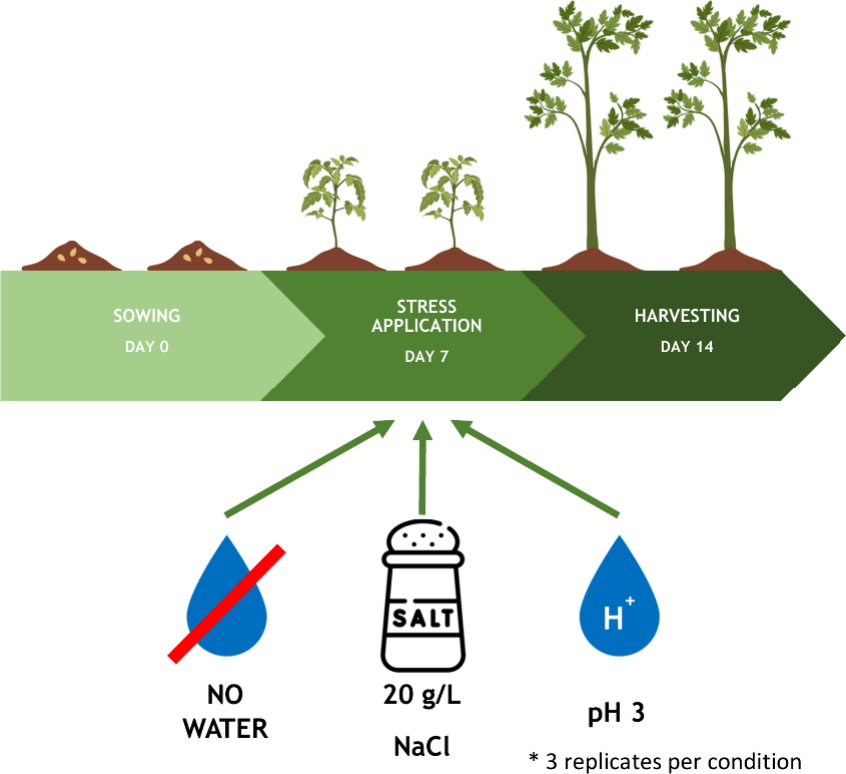
Schematic representation of the study design. Seeds of *Lepidium sativum* were sown on day 0. On day 7, three groups were formed and individually subjected to abiotic stress conditions for a duration of 7 days. Samples were harvested on day 14 for further analysis.

### Extraction and Analysis of Plant Metabolites

Samples of *Lepidium sativum* were initially separated into stems and leaves and then weighed (approximately 100 mg) in 2 mL microtubes; finally, 10 ceramic beads (1.4 mm) were added. IS were added to each sample at a final concentration of 2.5 ng/mg. For extraction, five volumes of a mixture of methanol:Milli-Q water:acetonitrile (50:30:20 v/v) were added (approximately 500 μL). Samples were homogenized using a Precellys Evolution (Bertin Instruments, France) for two cycles at 6800 rpm for 10 s, followed by centrifugation at 13,200 rpm for 10 min at 4 °C. Supernatants were collected for HPLC-HRMS analysis. The analyses were performed on a 1200 LC system (Agilent Technologies), coupled to an Orbitrap Q-Exactive mass spectrometer (Thermo Fisher), operating in ESI-positive and -negative ion full scan MS (70–1000 u) at a resolution of 35,000, and in data-dependent MS2 at 17,500 resolution (20–35–40 NCE collision energies). Chromatographic separation was performed on an Atlantis T3 column (2.1 mm × 150 mm, 3 μm particle size, Waters). For positive ionization mode analysis, a gradient of 0.1% formic acid (A) and acetonitrile (B) was applied (1% to 99% of B over 30 min, at a flow rate of 200 μL/min). For negative ionization mode analysis, a gradient of 5 mM ammonium formate in water (A) and acetonitrile (B) was used under the same conditions.

### Measurement of Plant Ecological Traits: Shoot Height, Biomass, and Number of Leaves

At the end of the experiment, shoot dry weight, shoot height, and leaf number were measured to investigate the effect of the selected stressors on plant growth. More specifically, shoot masses were measured with four decimal places after the samples were dried at 60 °C for 72 h, while shoot height was measured using a precision caliper. The significance of differences among the treatments was analyzed by one-way analysis of variance (ANOVA) or a Student's *t*-test using XLSTAT software. Prior to the analysis, the data were checked for normality and homogeneity of variances. Differences between treatments were detected, and mean values were compared by the Tukey’s test (*p* = 0.05).

### Data Analysis with Compound Discoverer Software for Unknown Metabolite Identification

Raw data obtained from HPLC-HRMS analysis were elaborated with Compound Discoverer 3.3 software (CD) (Thermo Fisher, Waltham, MA) for unknown metabolite identification. The workflow “Untargeted metabolomic with statistics detect unknowns with ID using online database and mzLogic” was modified (Table S9) as follows: the node “select spectra” parameter “total intensity threshold” was increased to 1,000,000 and “minimum peak count” set at 2. “Unrecognized MS resolution @200 replacement” and “Unrecognized MSn resolution @200 replacement” were, respectively, set to 35,000 and 17,500, i.e., the same resolution values used for the Orbitrap analysis. Based on the mode ionization, the “polarity mode” was set to “+” or “–”. In the following node “align retention time”, an intermediate file was selected. The node “detect compound” parameter “minimum peak intensity” was changed at 400,000 to make sure that peaks with low intensity were excluded. In parameter “ions”, for positive ionization, all the possible positive ions were selected, contrariwise, for negative ionization, all the possible negative ions were selected. Node “group compound” values of “number of files” and “peak rating threshold” were set, respectively, to 3 and 4, as suggested by the software. The node “search mzCloud” parameter “precursor mass tolerance” was set to 5 ppm. Value of “match activation energy” was changed in “any”. The node “predict composition” in the “S/N threshold” was set at 1.5, and “mass tolerance” was increased to 10 ppm. Node “Search Chemspider” was possible to select databases to use for compound research. For this study, the following databases were selected: BioCyc; Biosynth; Carotenoids database; Food and agriculture organization of the United Nations; FooDB; Human metabolome database; KEGG; MassBank; Nature chemical biology; Nature chemistry; NIST; NIST chemistry WebBook; NIST Spectra; and PubMed. In “Assign compound annotation,” the entry “data source #5” was changed with mzVault search, and “SFit range” set to 10. The node “Apply mzLogic” was changed to the entry “max # mzCloud similarity results to consider”, setting it to 30. The node “mark background compounds” in the ratio “max. sample/blank” was set to 2.

Statistical analysis was performed using Compound Discoverer Software, which performed a one-way ANOVA model, with Tukey as a post hoc test. *P*-values were adjusted by the Benjamini–Hochberg algorithm. Data were considered statistically significant with a *p*-value set <0.05 and log_2_ fold change >1. Data were also filtered out according to the following criteria:

1. Annotation Δmass [ppm] must be less than or equal to 6 ppm;
2. mzCloud best match must be more than or equal to 80;
3. Annotation source at least 4 “full matches out of 6”. The 6 annotations selected were Predicted Compositions, mzCloud, mzVault, Metabolika, Chemspider, and Mass list.

Metabolites that resulted significantly altered were imported into MetaboAnalyst 5.0 for the identification of metabolic pathway alterations, and “*Arabidopsis thaliana* *(thale cress) (KEGG)*” was set as the reference. Metabolic pathways meeting the following thresholds were considered as altered: −log­(*p*) > 0.05; FDR (false discovery rate) < 0.1, with a pathway coverage of at least two related metabolites. A table listing all of the changed parameters in the workflow can be found in the Supporting Information.

## Supplementary Material



## Data Availability

All metabolic data used in the present study are available in the online Supporting Information.

## Notes

List of all metabolites identified in *Lepidium sativum* leaf samples; list of all metabolites identified in *Lepidium sativum* stem samples; table listing all metabolites identified in *Lepidium sativum* leaf samples treated in drought conditions; table listing all metabolites identified in *Lepidium sativum* stem samples treated in drought conditions; table listing all metabolites identified in *Lepidium sativum* leaf samples treated with high-salinity water; table listing all metabolites identified in *Lepidium sativum* stem samples treated with high-salinity water; table listing all metabolites identified in *Lepidium sativum* leaf samples treated with acidified water; table listing all metabolites identified in *Lepidium sativum* stem samples treated with acidified water; and parameters changed in the Compound Discoverer workflow and their values (PDF)
