# Supplementary material for: Metabolic Responses of Plants to Climate-Induced Stress: A Mass Spectrometry Investigation
Source: ACS Omega. 2025 Jul 9;10(33):37342–52. doi: 10.1021/acsomega.5c02962 (PMC12392016; doi:10.1021/acsomega.5c02962)
Supplement: Supplementary file 1 [file ao5c02962_si_002.pdf]

Metabolic Responses of Plants to Climate-Induced Stress: A Mass Spectrometry Investigation

Matteo Preziati[1], Enrico Davoli[1], Antonio Di Guardo[2], Renzo Bagnati[1], Elisa Terzaghi[2]*, Alice Passoni[1]*

*These authors share co-last authorship

[1] Department of Environmental Health Sciences, Istituto di Ricerche Farmacologiche Mario Negri IRCCS, via Mario Negri 2, Milano

[2] Department of Science and High Technology (DiSAT), University of Insubria, Via Valleggio 11, 22100 Como, Italy

Corresponding author:

Alice Passoni

alice.passoni@marionegri.it

via Mario Negri 2, 20156 Milano

ORCID ID: 0000-0001-6003-5932

TABLE OF CONTENTS:

Supplementary Table 1: List of all metabolites identified in *Lepidium sativum* leaf samples. The green colored box indicates that the compound has been identified significantly altered in the sample. The box-colored red indicates that the metabolite was not identified significantly altered in the sample.

Supplementary Table 2: List of all metabolites identified in *Lepidium sativum* stem samples. The green colored box indicates that the compound has been identified in the sample. The box-colored red indicates that the metabolite was not identified in the sample

Supplementary Table 3: Table listing all metabolites identified in Lepidium sativum leaf samples treated in drought condition. Metabolites shown in green were up-regulated, while those in red were down-regulated.

Supplementary Table 4: Table listing all metabolites identified in Lepidium sativum stem samples treated in drought condition. Metabolites shown in green were up-regulated, while those in red were down-regulated.

Supplementary Table 5: Table listing all metabolites identified in Lepidium sativum leaf samples treated with high-salinity water. Metabolites shown in green were up-regulated, while those in red were down-regulated.

Supplementary Table 6: Table listing all metabolites identified in Lepidium sativum stem samples treated with high-salinity water. Metabolites shown in green were up-regulated, while those in red were down-regulated.

Supplementary Table 7: Table listing all metabolites identified in Lepidium sativum leaf samples treated with acidified water. Metabolites shown in green were up-regulated, while those in red were down-regulated.

Supplementary Table 8: Table listing all metabolites identified in Lepidium sativum stem samples treated with acidified water. Metabolites shown in green were up-regulated, while those in red were down-regulated.

Supplementary Table 9: Parameters changed in the Compound Discoverer workflow and their values

Supplementary Table 1: List of all metabolites identified in *Lepidium sativum* leaf samples. The green-colored box indicates that the compound was identified significantly altered in the sample, while the red-colored box indicates that the metabolite was not identified significantly altered in the sample.

Metabolite	pH	Salinity	Drought
(+/-)-Gingerol	Green	Green	Green
D-(+)-Tryptophan	Green	Green	Green
L-Phenylalanine	Green	Green	Green
L-Saccharopine	Green	Green	Green
1-Linoleoyl glycerol	Red	Green	Green
D-(+)-Proline	Red	Green	Green
L-2-Aminoadipic acid	Red	Green	Green
N-Phenylacetylglutamine	Red	Green	Green
Proline	Red	Green	Green
Syringic acid	Red	Green	Green
Leucylproline	Green	Red	Green
L-Histidine	Green	Red	Green
2-Amino-4-methylpyrimidine	Red	Red	Green
2'-O-Methyladenosine	Red	Red	Green
4-Coumaric acid	Red	Red	Green
4-Guanidinobutyric acid	Red	Red	Green
4-Hydroxybenzoic acid	Red	Red	Green
9-Oxo-10(E),12(E)-octadecadienoic acid	Red	Red	Green
Adenine	Red	Red	Green
Cytarabine	Red	Red	Green
Cytosine	Red	Red	Green
Tyrosylalanine	Red	Red	Green
Valine	Red	Red	Green
Valylproline	Red	Red	Green
α-Aspartylphenylalanine	Red	Red	Green
α-Lactose	Red	Red	Green
Y-L-Glutamyl-L-glutamic acid	Red	Red	Green
L-Lysine	Red	Red	Green
D-Carnitine	Red	Red	Green
D-Cysteine	Red	Red	Green
DL-Arginine	Red	Red	Green
Glucose 1-phosphate	Red	Red	Green
Glycylproline	Red	Red	Green
Guanine	Red	Red	Green
Imidazolelactic acid	Red	Red	Green
Jasmonic acid	Red	Red	Green
L-(-)-Methionine	Red	Red	Green
L-Glutamic acid	Red	Green	Red
L-Methionine sulfoxide	Red	Green	Red
L-Threonine	Red	Green	Red
N-Acetylornithine	Red	Green	Red
Syringic acid	Red	Green	Red
D-(-)-Aspartic acid	Red	Green	Red

Supplementary Table 2: List of all metabolites identified in *Lepidium sativum* stem samples. The green-colored box indicates that the compound was identified significantly altered in the sample, while the red-colored box indicates that the metabolite was not identified significantly altered in the sample.

Metabolite	pH	Salinity	Drought
Syringic acid	green	green	green
(+/-)-Gingerol	red	green	green
12-Oxo phytodienoic acid	red	green	green
2'-O-Methyladenosine	red	green	green
3,4-MDPA	red	green	green
Cytarabine	red	green	green
Cytosine	red	green	green
D-(-)-Aspartic acid	red	green	green
D-(+)-Proline	red	green	green
9-Oxo-10(E),12(E)-octadecadienoic acid	red	green	green
DL-Serine	red	green	green
DL-Tryptophan	red	green	green
Glycylproline	red	green	green
Guanine	red	green	green
L-2-Aminoadipic acid	red	green	green
Leucylproline	red	green	green
L-Lysine	red	green	green
L-Phenylalanine	red	green	green
L-Saccharopine	red	green	green
L-Threonine	red	green	green
Nicotinamide	red	green	green
N-Phenylacetylglutamine	red	green	green
N-α-L-Acetyl-arginine	red	green	green
Tyrosylalanine	red	green	green
N6,N6,N6-Trimethyl-L-lysine	red	red	green
4-Guanidinobutyric acid	red	red	green
4-Hydroxybenzoic acid	red	red	green
5'-S-Methyl-5'-thioadenosine	red	red	green
Adenine	red	red	green
Adenosine	red	red	green
L-Pyroglutamic acid	red	red	green
1-Linoleoyl glycerol	red	red	green
2-Amino-4-methylpyrimidine	red	red	green
D-Cysteine	red	red	green
DL-Arginine	red	red	green
Glucose 1-phosphate	red	red	green
Jasmonic acid	red	red	green
L-Histidine	red	red	green
Sinapinic acid	red	red	green
tert-Butyl N-[1-(aminocarbonyl)-3-methylbutyl]carbamate	red	red	green
Valine	red	red	green
Valylproline	red	red	green
α-Aspartylphenylalanine	red	red	green
α-Lactose	red	red	green
Y-L-Glutamyl-L-glutamic acid	red	red	green
N-Acetyl-L-leucine	red	green	red
N-Benzylformamide	red	green	red
DL-Glutamine	red	green	red
Ferulic acid	red	green	red
L-Methionine sulfoxide	red	green	red
4-Coumaric acid	red	green	red
Coniine	green	red	red

Supplementary Table 3: Table listing all metabolites identified in Lepidium sativum leaf samples treated in drought condition. Metabolites shown in green were up-regulated, while those in red were down-regulated.

Metabolite	Drought
(+/-)-Gingerol	
1-Linoleoyl glycerol	
2'-O-Methyladenosine	
2-Amino-4-methylpyrimidine	
4-Coumaric acid	
4-Guanidinobutyric acid	
4-Hydroxybenzoic acid	
9-Oxo-10(E),12(E)-octadecadienoic acid	
Adenine	
Cytarabine	
Cytosine	
D-(+)-Tryptophan	
D-Carnitine	
D-Cysteine	
DL-Arginine	
Glucose 1-phosphate	
Glycylproline	
Guanine	
Imidazolelactic acid	
Jasmonic acid	
L-(-)-Methionine	
L-2-Aminoadipic acid	
L-Histidine	
L-Lysine	
L-Phenylalanine	
L-Saccharopine	
Leucylproline	
N-Phenylacetylglutamine	
Proline	
Syringic acid	
Tyrosylalanine	
Valine	
Valylproline	
Y-L-Glutamyl-L-glutamic acid	
α-Aspartylphenylalanine	
α-Lactose	

Supplementary Table 4: Table listing all metabolites identified in Lepidium sativum stem samples treated in drought condition. Metabolites shown in green were up-regulated, while those in red were down-regulated.

Metabolite	Drought
Valylproline	
D-(+)-Proline	
DL-Tryptophan	
DL-Arginine	
α-Lactose	
Glycylproline	
3,4-MDPA	
Glucose 1-phosphate	
2-Amino-4-methylpyrimidine	
2'-O-Methyladenosine	
L-Histidine	
L-Phenylalanine	
L-Saccharopine	
L-Pyroglutamic acid	
Leucylproline	
4-Guanidinobutyric acid	
Valine	
N-Phenylacetylglutamine	
9-Oxo-10(E),12(E)-octadecadienoic acid	
Syringic acid	
L-2-Aminoadipic acid	
Adenine	
Guanine	
Cytosine	
Cytarabine	
Tyrosylalanine	
N6,N6,N6-Trimethyl-L-lysine	
1-Linoleoyl glycerol	
Nicotinamide	
(+/-)-Gingerol	
N-α-L-Acetyl-arginine	
5'-S-Methyl-5'-thioadenosine	
α-Aspartylphenylalanine	
Y-L-Glutamyl-L-glutamic acid	
Sinapinic acid	
4-Hydroxybenzoic acid	
L-Lysine	
D-Cysteine	
tert-Butyl N-[1-(aminocarbonyl)-3-methylbutyl]carbamate	
Jasmonic acid	
Adenosine	
12-Oxo phytodienoic acid	
L-Threonine	
DL-Serine	
D-(-)-Aspartic acid	

Supplementary Table 5: Table listing all metabolites identified in Lepidium sativum leaf samples treated with high-salinity water. Metabolites shown in green were up-regulated, while those in red were down-regulated.

Metabolite	Salinity
L-Methionine sulfoxide	
N-Phenylacetylglutamine	
N-Acetylornithine	
DL-Tryptophan	
L-Phenylalanine	
Syringic acid	
Proline	
L-2-Aminoadipic acid	
L-Saccharopine	
1-Linoleoyl glycerol	
(+/-)-Gingerol	
L-Threonine	
D-(-)-Aspartic acid	
L-Glutamic acid	

Supplementary Table 6: Table listing all metabolites identified in Lepidium sativum stem samples treated with high-salinity water. Metabolites shown in green were up-regulated, while those in red were down-regulated.

Metabolite	Salinity
3,4-MDPA	
L-Methionine sulfoxide	
L-Saccharopine	
2'-O-Methyladenosine	
N-Phenylacetylglutamine	
N-Acetyl-L-leucine	
Guanine	
D-(+)-Proline	
Glycylproline	
9-Oxo-10(E),12(E)-octadecadienoic acid	
Cytarabine	
Cytosine	
Leucylproline	
L-Phenylalanine	
(+/-)-Gingerol	
DL-Tryptophan	
L-2-Aminoadipic acid	
4-Coumaric acid	
Tyrosylalanine	
N-α-L-Acetyl-arginine	
Ferulic acid	
N-Benzylformamide	
Syringic acid	
Nicotinamide	
L-Lysine	
12-Oxo phytodienoic acid	
L-Threonine	
DL-Glutamine	
DL-Serine	
D-(-)-Aspartic acid	

Supplementary Table 7: Table listing all metabolites identified in Lepidium sativum leaf samples treated with acidified water. Metabolites shown in green were up-regulated, while those in red were down-regulated.

Metabolite	pH
DL-Tryptophan	
Leucylproline	
Syringic acid	
(+/-)-Gingerol	
L-Saccharopine	
L-Phenylalanine	
L-Histidine	

Supplementary Table 8: Table listing all metabolites identified in Lepidium sativum stem samples treated with acidified water. Metabolites shown in green were up-regulated, while those in red were down-regulated.

Metabolite	pH
Syringic acid	
Coniine	

Supplementary Table 9: Parameters modified in the Compound Discoverer workflow and their corresponding values.

Node	Parameter	Value
select spectra	total intensity threshold	1000000
	minimum peak count	2
	Unrecognized MS resolution @200 replacement	35000
	Unrecognized MSn resolution @200 replacement	17500
	polarity mode	+ (for positive ionization); - (for negative ionization)
align retention time	reference file	an intermediate file was selected
detect compound	minimum peak intensity	400000
	ions	for positive ionization all positive ions were selected; for negative ionization all negative ions were selected
group compound	number of files	3
	peak rating threshold	4
search mzCloud	precursor mass tolerance	5 ppm
	match activation energy	any
predict composition	S/N threshold	1.5
	mass tolerance	10 ppm
Search Chemspider	databases	BioCyc; Biosynth; Carotenoids database; Food and agriculture organization of the United Nations; FooDB; Human metabolome database; KEGG; MassBank; Nature chemical biology; Nature chemistry; NIST; NIST chemistry WebBook; NIST Spectra; PubMed
Assign compound annotation	data source #5	mzVault
	SFit range	10
Apply mzLogic	max # mzCloud similarity results to consider	30
mark background compounds	max. sample/blank	2
